# Intronic Single Nucleotide Polymorphisms in FGFR2 Gene Association With Non‐Syndromic Mandibular Retrognathism

**DOI:** 10.1111/ocr.70081

**Published:** 2026-01-12

**Authors:** Caio Luiz Bitencourt Reis, Christian Kirschneck, Daniel Hemming, Eva Paddenberg‐Schubert, Peter Proff, Daniela Silva Barroso de Oliveira, Cristiano Miranda de Araujo, Flares Baratto‐Filho, Svenja Beisel‐Memmert, Erika Calvano Küchler

**Affiliations:** ^1^ School of Dentistry Federal University of Alfenas Alfenas Minas Gerais Brazil; ^2^ Department of Orthodontics University Hospital Bonn Bonn Germany; ^3^ School of Dentistry Tuiuti University of Paraná Curitiba Parana Brazil; ^4^ Department of Orthodontics University of Regensburg Regensburg Germany; ^5^ School of Dentistry University of the Joinville Region Joinville Santa Catarina Brazil

**Keywords:** fgfr2, mandible, mandibular retrognathism, orthodontic, single nucleotide polymorphism

## Abstract

**Objective:**

Mandibular retrognathism (MR) is a skeletal malocclusion in which patients have a deficient mandibular length, resulting in a more posterior position of the mandible. We aimed to investigate the association between Single nucleotide polymorphisms (SNPs) in *Fibroblast Growth Factor Receptor 2* (*FGFR2)* gene and MR in germans.

**Materials and Methods:**

Genomic DNA and lateral cephalometric radiographs were obtained from orthodontic patients. Patients were allocated into the ‘Retruded’ group (SNB angle < 78°) and into the ‘Well‐positioned’ group (SNB 78°–82°). The rs4752566, rs10736303, rs11200014, rs1078806, rs1219648, rs2981578 and rs2162540 SNPs were genotyped using real‐time PCR. Allele, genotype and haplotype frequencies were compared (α = 5%).

**Results:**

A total of 142 patients were included, 93 (65.5%) allocated into the ‘Retruded’ group and 49 (34.5%) into the ‘Well‐positioned’ group. The allele T in rs2981578 SNP was statistically more frequent in the ‘Retruded’ group in both univariate (PR = 1.22; 95% CI, = 1.02–1.47) and multivariate (PR = 1.55; 95% CI, = 1.07–2.25) analyses (*p* < 0.05). The CT + TT genotypes were statistically more frequent in the ‘Retruded’ group in univariate (PR = 1.58; 95% CI, = 1.03–2.41) and multivariate (PR = 1.59; 95% CI, = 1.11–2.26) analysis (*p* < 0.05). All studied SNPs were associated with MR establishment in haplotype analysis (*p* < 0.05).

**Conclusion:**

SNPs in the *FGFR2* are associated with MR and have the potential to serve as genetic biomarkers to early diagnosis and prediction of mandible growth.

## 1 Introduction

Mandibular retrognathism (MR) or retrognathia is the predominant condition related to skeletal Class II malocclusion [1, 2, 3]. Class II malocclusion is the second most prevalent anteroposterior malocclusion in Europe, affecting 36.7% of the population during permanent dentition [4]. Patients with MR have a deficient mandibular length, resulting in a more posterior position of the mandible in relation to the maxilla and cranial base. Patients often exhibit a convex profile, chin retrusion, accentuated axial inclination of lower incisors and large overjet [5]. The MR etiology is multifaceted, influenced by an interplay of both environmental and genetic factors. Recent studies have highlighted the impact of genetic variations on craniofacial growth in individuals with MR [6, 7, 8]. A comprehensive investigation of these variations is important for early diagnosis, accurate individual growth prediction and to develop novel orthodontic and orthognathic treatment approaches [9].

The fibroblast growth factor receptor 2 (FGFR2) is a member of the receptor tyrosine kinase family (FGFR 1–4) and interacts with over 20 proteins ligands [10]. FGFR2 is a growth factor essential in the regulation of bone growth and the development of the craniofacial skeleton starting from the embryonic stage. It controls the proliferation and differentiation of osteoblasts, the cells responsible for bone formation [11]. FGFR2 has two isoforms due to mRNA alternative splicing, known as FGFR2b and FGFR2c. The FGFR2c isoform, also known as Bek/FGFR2, is primarily found in cranial bones, maxilla and mandible [12]. Mutations in the *FGFR2* gene have been linked to craniosynostosis syndromes, including Apert, Crouzon and Pfeiffer syndromes. These syndromes are characterised by abnormal maxillomandibular discrepancies [13, 14]. Genetic variations in *FGFR2* have also been studied in non‐syndromic malocclusion, especially mandibular prognathism [15, 16, 17, 18], but its association MR have also been proposed [15, 18, 19].

Single nucleotide polymorphisms (SNPs) are the most prevalent form of genetic variation across populations. These variations involve the alteration of a single nucleotide within a specific genomic region. Intronic SNPs are situated within the non‐coding region of a gene, thus not affecting the amino acid sequence of the protein. Nevertheless, they can influence gene transcription and posttranscriptional processes, such as microRNA excision [20, 21]. A recent systematic review [19] suggests the need for further studies to elucidate the impact of intronic SNPs in *FGFR2* on the establishment of non‐syndromic skeletal malocclusions. The identification of these SNPs could provide insights into the aetiology of MR and potentially serve as biomarkers for early diagnosis and individual growth prediction. Thus, this study aimed to investigate the potential impact of intronic SNPs on MR establishment in germans orthodontic patients.

## 2 Methods

### 2.1 Ethics Statement

The sample analysed in this current cross‐sectional study was previously described by Kischneck et al. (2022), [7] as well as the sample size calculation. No additional patients were added to the sample after the initial study. The Human Ethics Committee at the University of Regensburg, Germany, granted approval for this study (reference number 19–1549‐101). All ethical guidelines outlined in the Helsinki Declaration were followed. Informed consent was obtained from all patients and their parents or legal guardians. Additionally, for patients under the age of 14, an age‐appropriate assent document was also utilised.

### 2.2 Study Design

The study followed the Strengthening the Reporting of Genetic Association Studies (STREGA) guideline, which is an extension of the STROBE Statement [22].

### 2.3 Setting and Participants

Orthodontic patients from private clinics in Regensburg City and the Department of Orthodontics at the University of Regensburg were screened between 2020 and 2021. We recruited patients of both sexes, aged 7–18 years, who were in the mixed and permanent dentition stages. We excluded patients with syndromes, adults over 18 years old, congenital alterations including tooth agenesis (except for third molar agenesis), oral cleft patients, those with a history of previous orthodontic treatment and patients with facial trauma. Additionally, patients with mandibular prognathism, a biological relationship with an already included patient and those with unsuccessful genotyping for all studied SNPs were also excluded.

### 2.4 Variables and Data Sources

MR was diagnosed according to the method proposed by Steiner [23]. Cephalometric analysis was conducted using digital lateral cephalograms through Ivoris software (Computer Konkret AG, Falkenstein, Germany, version 8.2.15.110). Two trained and calibrated examiners manually determined the SNB angle (the posteroinferior angle formed by the sella–nasion and nasion–B point planes). Patients were allocated into the ‘Retruded’ group if SNB < 78° and the ‘Well‐positioned’ group if SNB ranged from 78° to 82°. The SNB was also evaluated as a continuous variable. Additionally, sex and age data were recorded for analysis.

Buccal epithelial cells were collected from each patient for genomic DNA extraction. Two cytobrushes were used to collect the cells, which were then placed in extraction solution (Tris–HCl, 10 mmol/L, pH 7.8; EDTA, 5 mmol/L and SDS, 0.5%; 1 mL). Next, proteinase K (100 ng/mL) was added to each tube, followed by the addition of ammonium acetate to remove non‐digested proteins. The resulting solution was then centrifuged. DNA was precipitated using isopropanol and washed with ethanol. The quantity and quality of DNA extracted were determined using spectrophotometry (Nanodrop 1000; Thermo Scientific, Wilmington, DE, USA).

Seven intronic SNPs in the *FGFR2* gene were selected according to their potential clinical relevance pointed out by previous studies (ncbi.nlm.nih.gov/snp). Details of the selected SNPs are shown in Table 1. The SNPs were genotyped using real‐time polymerase chain reaction (PCR) with the Mastercycler ep realplex‐S thermocycler (Eppendorf AG, Hamburg, Germany) and TaqMan technology. The process involved an initial denaturation at 95°C for 30 s, followed by 40 cycles of denaturation at 92°C for 5 s and annealing/extension at 60°C for 20 s. Each 3.125‐μL reaction volume contained 1.5 μL Master Mix, 0.125 μM TaqMan probe and 4 ng DNA in 1.5 μM nuclease‐free water. The assays and reagents were provided by Applied Biosystems (Foster City, CA, USA).

**TABLE 1:** Details about the selected SNPs in *FGFR2* gene.

Name	Position	Hardy–Weinberg equilibrium *p*	Genotype success rate	MAF	Alleles
rs4752566	121 508 117	0.555	95.6	0.412	G > T
rs10736303	121 574 943	0.349	99.3	0.404	A > G
rs11200014	121 575 416	0.557	95.6	0.351	G > A
rs1078806	121 579 461	> 0.999	99.3	0.390	A > G
rs2981578	121 580 797	0.308	93.4	0.496	C > T
rs1219648	121 586 676	0.969	100.0	0.358	A > G
rs2162540	121 592 622	0.110	100.0	0.401	T > C

*Note:* All SNPs are intronic variants. Hardy–Weinberg equilibrium *p*‐value was evaluated by chi‐square test.

Abbreviation: MAF, Minor Allele Frequency in this study.

### 2.5 Bias

To avoid information bias, intra‐ and inter‐examiner reliability were measured. The SNB angle of 20 additional patients not included in the original study was obtained to conduct a concordance test between the two examiners. Inter‐examiner reliability, as indicated by the intra‐class correlation coefficients (ICC), demonstrated excellent agreement for both examiners (ICC = 0.95). Intra‐examiner reliability was assessed by repeating the concordance test after a two‐week interval, which also showed excellent agreement (ICC = 0.91). Besides that, a negative control template was included in each genotype, and 10% of the samples were randomly selected for repeated analysis, which showed 100% concordance.

In order to prevent observation bias, both cephalometric examiners and professionals conducting molecular and statistical analyses were blinded to group, age and sex. Additionally, to minimize confounding bias, multivariate statistical analysis was performed including sex and age as co‐variables due to their known influence on MR establishment.

### 2.6 Statistical Methods

The Hardy–Weinberg equilibrium was evaluated by Pearson's Chi‐square test without correction. The same test was used to compare the frequency distribution (univariate analysis) among ‘Retruded’ and ‘Well‐positioned’ groups and alleles (Major allele vs. Minor allele) and genotypes. Two genotype comparison models were adopted: Dominant (Homozygous common vs. Heterozygous + Homozygous uncommon) and Recessive (Homozygous common + Heterozygous vs. Homozygous uncommon) models. Multivariate analysis adjusted by sex and age was analysed using Poisson regression. Prevalence ratio (PR) with 95% Confidence Interval (CI) was calculated in uni‐ and multivariate analysis when *p* < 0.05.

Haplotype analysis for the SNPs was conducted using PLINK version 1.06 (https://zzz.bwh.harvard.edu/plink/ld.shtml) and a linkage disequilibrium plot was generated by Haploview 4.1 [24].

The Mann–Whitney *U* test was used to compare continuous data (age and SNB angles) among the groups after assessing normality using the Shapiro–Wilk test. Median and interquartile range (IR) were calculated. Effect size was estimated using Cohen's D with 95% CI, and box‐plots were generated when *p* < 0.05. The relationship between age and continuous SNB variable was assessed using the Spearman test.

All tests were carried out using IBM SPSS Statistics for Windows (Version 25.0. Armonk, NY: IBM Corp.) For all analyses, the established alpha was *p* < 0.05.

## 3 Results

### 3.1 Sample Characteristics

The 146 patients initially included in the study were screened, with four patients excluded because their genetic information in the FGFR2 gene could not be successfully identified (genotyping failure). Thus, 142 patients were included in this current study, 74 males (52.1%) and 68 females (47.9%). The average age was 11.9 ± 5.00. Ninety‐three (65.5%) patients were allocated into the ‘Retruded group’ (SNB < 78°), and 49 (34.5%) were allocated into the ‘Well‐positioned group’ (SNB = 78°–82°). The distribution of males and females between the two groups was not statistically different (*p* = 0.220). The age was statistically lower in the ‘Retruded group’ (Median = 11.3; IR = 10.0–12.8) compared to the ‘Well‐positioned group’ (Median = 12.5; IR = 10.7–14.2) (*p* = 0.004). Age was also correlated with the continuous SNB variable (*R* = 0.234; *p* = 0.004). More details about the sample characteristics are shown in (Table 2). The raw data is shown in Table S1.

**TABLE 2:** Characteristics of the sample.

	Total	Well‐positioned	Retruded	*p*	SNB	*p*
	*n* (%)	*n* (%)	*n* (%)		median (IR)	
Total	142 (100)	49 (34.5)	93 (65.5)	—	76.6 (74.3–78.7)	—
Male	74 (52.1)	29 (59.2)	45 (48.4)	0.220	76.8 (74.4–79.4)	0.263
Female	68 (47.9)	20 (60.8)	48 (51.6)		76.4 (74.2–78.2)	
	Median (IR)			*p*	R	*p*
Age	11.8 (10.1–13.2)	12.5 (10.7–14.2)	11.3 (10.0–12.8)	0.004*	0.234	0.004*

*Note:* Sex was compared by chi‐square test and age by Mann–Whitney test between well‐positioned. and retruted groups. The SNB medians was compared also by Mann–Whitney test between sex. The correlation between age and SNB was estimated by Spearman correlation test.

* Means *p* < 0.05.

### 3.2 SNPs Characteristics

All studied SNPs in the *FGFR2* gene were in Hardy–Weinberg equilibrium (*p* > 0.05), and the genotype success rate ranged from 93.4% to 100%. Details about the Hardy–Weinberg equilibrium and genotype success rate are demonstrated in Table 1.

### 3.3 rs2981578 SNP Association With MR

The uni‐ and multivariate analysis among groups and SNPs are shown in Table 3. The rs2981578 SNP in the *FGFR2* gene was statistically associated with MR establishment in allelic and dominant models, both in uni‐ and multivariate tests (*p* < 0.05). The allele T of this SNP was statistically more frequent in the ‘Retruded group’ in both uni‐ (*p* = 0.023; PR = 1.22; 95% CI, = 1.02–1.47) and multivariate (*p* = 0.018; PR = 1.55; 95% CI, = 1.07 to 2.25) analysis. Besides, the genotypes CT and TT were statistically more frequent in the ‘Retruded group’ in uni‐ (*p* = 0.009; PR = 1.58; 95% CI, = 1.03–2.41) and multivariate (*p* = 0.010; PR = 1.59; 95% CI, = 1.11–2.26). No statistical difference among groups was found for the other studied SNPs (*p* > 0.05).

**TABLE 3:** Genotype comparison per models between well‐positioned and retruded groups.

SNPs	Genotype	Well‐positioned		Retruded		Allelic		Dominant		Recessive	
		*N*	%	*N*	%	*p* ^u^	*p* ^m^	*p* ^u^	*p* ^m^	*p* ^u^	*p* ^m^
rs4752566	GG	17	35.42	28	32.56	0.254	0.626	0.736	0.341	0.080	0.086
	GT	27	56.25	41	47.67						
	TT	4	8.33	17	19.77						
rs10736303	AA	12	24.49	33	37.50	0.105	0.450	0.120	0.165	0.255	0.533
	AG	28	57.14	45	51.14						
	GG	9	18.37	10	11.36						
rs11200014	GG	18	37.50	35	42.17	0.376	0.335	0.599	0.467	0.272	0.313
	AG	23	47.92	41	49.40						
	AA	7	14.58	7	8.43						
rs1078806	AA	14	28.57	37	42.05	0.480	0.112	0.117	0.152	0.454	0.739
	AG	29	59.18	36	40.91						
	GG	6	12.24	15	17.05						
rs2981578	CC	16	35.56	13	15.48	0.023*	0.018*	0.009*	0.010*	0.214	0.163
	CT	22	48.89	50	59.52						
	TT	7	15.56	21	25.00						
rs1219648	AA	17	34.69	40	45.45	0.193	0.552	0.220	0.362	0.409	0.467
	AG	24	48.98	38	43.18						
	GG	8	16.33	10	11.36						
rs2162540	TT	15	30.61	32	34.41	0.266	0.112	0.647	0.706	0.088	0.183
	CT	25	51.02	53	56.99						
	CC	9	18.37	8	8.60						

*Note:* Univariate *p*‐value was obtained by chi‐square test. Multivariate *p*‐value was obtained by Poisson Regression adjusted by age and gender.

^u^ Means univariate test.

^m^ Means multivariate test.

* Means *p* < 0.05.

**FIGURE 1:**
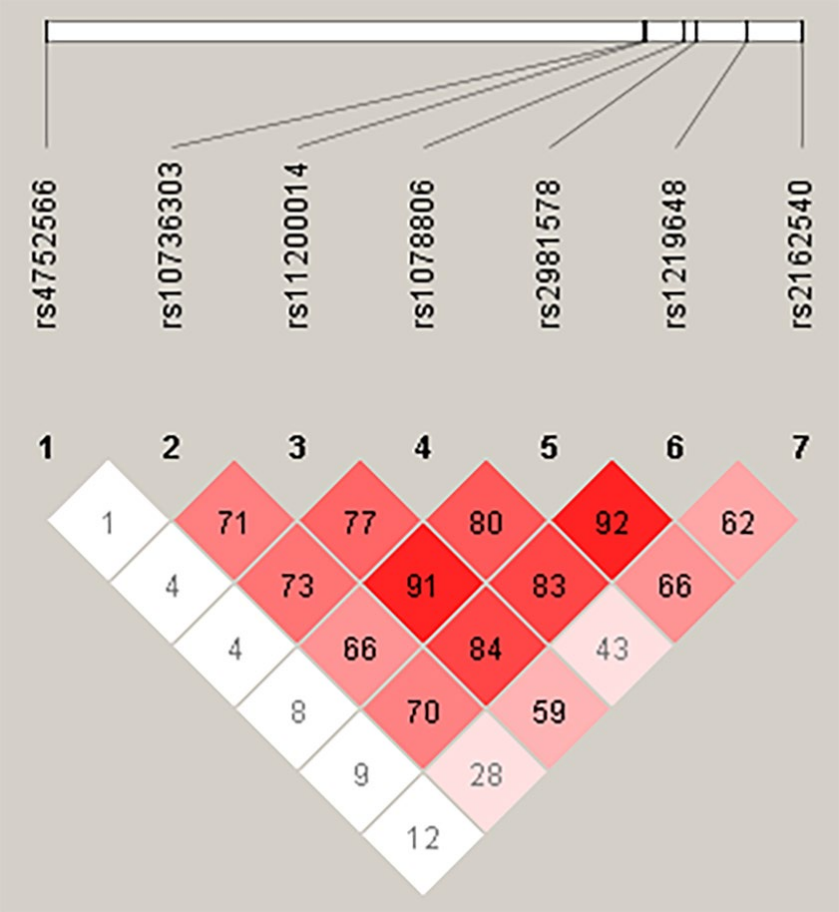
Box‐plot comparing SNB angle values among rs2981578 genotypes (CC, CT and TT). The box represents the interquartile range (IQR) and whiskers indicate 1.5 × IQR. The median SNB angle was lower in carriers of the T allele.

The rs2981578 SNP impact on MR establishment was also evaluated as a continuous SNB variable. SNB values in patients with heterozygous CT (Median = 76.1; IR = 74.1–78.3; Cohen's d −0.56 95% CI, = −1.00–0.12; *p* < 0.001) and uncommon homozygous TT (Median = 76.1; IR = 72.9–78.3; Cohen's d −0.66 95% CI, = 1.2 –0.13; *p* < 0.001) were statistically lower compared to patients with common homozygous CC (Median = 78.0; IR = 75.7–79.4). (Figure 1) illustrates the distribution of SNB values according to rs2981578 genotypes, highlighting that carriers of the T allele presented lower mandibular projection.

### 3.4 Haplotype Analysis

**FIGURE 2:**
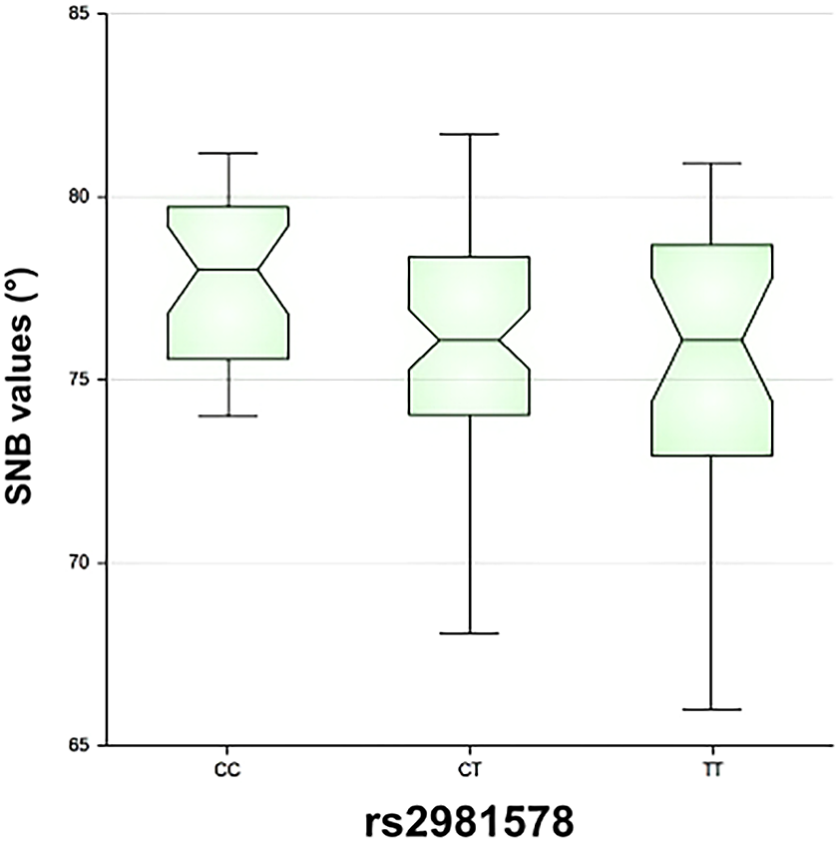
Linkage disequilibrium (LD) plot. The colour intensity represents the degree of pairwise LD between SNPs, expressed as *r* ^2^ values. Darker colours indicate stronger LD, suggesting that these intronic variants are inherited together and may jointly contribute to MR susceptibility.

The haplotype analysis examined 882 different haplotype combinations, which are demonstrated in Table S2. Overall, all studied SNPs were associated with MR establishment in haplotype analysis (*p* < 0.05). Table 4 reports the 13 statistically associated haplotype combinations (*p* < 0.05). The linkage disequilibrium plot (Figure 2) between the combinations indicated in the Table 4 demonstrates a strong linkage between several studied loci.

**TABLE 4:** Haplotype analysis.

SNPs combination (bigger to smaller association)	Allele combination	Frequency in controls	Frequency in cases	*x* ^2^	*p*
rs2981578|rs10736303	TA	0.3128	0.492	7.649	0.005
rs2981578|rs10736303|rs1219648	TAA	0.3284	0.4884	6.498	0.010
rs2981578|rs10736303|rs11200014	TAG	0.3295	0.4744	5.395	0.020
rs2981578|rs4752566^b^	TT	0.1442	0.2655	4.813	0.028
rs2162540|rs2981578|rs10736303	TTA	0.2767	0.4051	4.528	0.033
rs2981578|rs4752566|rs1219648	TTA	0.1448	0.2533	4.386	0.036
rs2162540|rs2981578|rs4752566	CCG	0.2762	0.1709	4.29	0.038
rs2162540|rs2981578|rs10736303|rs11200014	TTAG	0.2806	0.4048	4.17	0.041
rs2162540|rs4752566|rs10736303|rs1078806|rs1219648	CGGGG	0.2438	0.1455	4.069	0.043
rs2162540|rs2981578|rs10736303|rs11200014|rs1219648	TTAGA	0.2872	0.412	4.01	0.045
rs4752566|rs10736303^a^	GG	0.3087	0.1998	3.962	0.046
rs2981578|rs1219648^b^	TA	0.3978	0.5276	3.936	0.047
rs2981578|rs10736303|rs1078806	TAA	0.3095	0.4316	3.905	0.048

*Note:* 882 different combinations of haplotypes were generated and compared by PLINK. Only the relevant *p* < 0.05 combinations are reported here.

^a^ Means high linkage disequilibrium.

^b^ Means low linkage disequilibrium.

## 4 Discussion

Recent studies demonstrated that MR has a significant impact on various aspects of patients’ lives, including their quality of life [25], self‐esteem [26], masticatory function and airway [14]. Patients with MR often require orthodontic and, occasionally, orthognathic surgery. However, the success of these treatments is strongly associated with early diagnosis and accurate individual growth prediction [13, 14]. Exploring the impact of SNPs on MR establishment may identify relevant genetic markers to assist orthodontists in early identification of individuals at higher risk for MR, besides predicting individual remanescent growth, individualising treatment and reducing potential side effects, such as relapse, treatment failure and temporomandibular dysfunction. In this study, we found an association between the seven intronic SNPs in *the FGFR2* gene and MR in a german population, thereby rejecting the null hypothesis. These findings suggest that genetic screening for *FGFR2* variants could complement the clinical orthodontic practice.

A recently scoping review showed a hypothesis‐generating research analysing the shared chromosomal loci and/or common genetic mechanisms and molecular pathways that may be involved in the occurrence of syndromes with the skeletal malocclusion phenotypes, highlighting *FGFR2* as a gene possibly involved in both syndromic and non‐syndromic skeletal malocclusion [27]. The *FGFR2* gene was the focus of our study also due to its involvement in multiple signalling pathways that regulate cell proliferation, differentiation, migration and apoptosis during craniofacial bone development. The gene is located on chromosome 10 and produces at least two different receptor isoforms, FGFR2b and FGFR2c. The receptors differ in the binding domain due to alternative splicing of exon 8 in FGFR2b and exon 9 in FGFR2c. The c isoform, or Bek, is significantly expressed during osteogenesis, regulating chondrocytes, osteocytes and osteoblasts proliferation, differentiation and function [10, 11, 12]. Genetic disorders associated with mutations in *FGFR2*, such as craniosynostosis syndromes (e.g., Crouzon, Apert and Pfeiffer) exhibit similar phenotypes characterised by premature fusion of the craniofacial sutures and significant retrusion of the midface [13, 14]. Investigation of genetic variations, such as SNPs, in *FGFR2* has also been conducted in non‐syndromic malocclusions, particularly maxillary retrusion [19]. SNPs in *FGFR2* have been associated with MR in previous studies, corroborating with our current findings [6, 8]. This study reinforces the hypothesis that *FGFR2* acts as a regulatory gene in craniofacial growth and that genetic variations may affect its expression or function.

Despite the main growth centre of the mandible being the condyle, an endochondral growth region [28], chondrocytes are sensitive to *FGFR2* regulation [29]. Besides that, the process of substitution of cartilage by bone is executed by osteoblasts. Lastly, the mandibular ramus is an important intramembranous growth centre responsible for the forward direction growth of the mandible [28]. In this way, SNPs in FGFR2 may be risk factors for several skeletal malocclusions.

In this study, the rs2981578 SNP in the *FGFR2* gene was significantly associated with MR in both uni‐ and multivariate analyses. This particular SNP has been extensively studied and is frequently linked to both non‐syndromic and syndromic craniosynostosis according to the dbSNP database. An in vitro study [30] showed that the recessive allele of the rs2981578 SNP (T) functions as a transcriptional silencer element, triggering the silencer activity of the FGFR2 gene and decreasing gene expression. Furthermore, an animal study [31] involving an Fgfr2 conditional knockout lineage of mice exhibited a significant reduction in mandible development during embryonic stages. These findings indicated the essential role of Fgfr2 in maintaining mesenchymal FGFs signalling to regulate osteoblast differentiation during mandibular development. Based on these studies, we hypothesised that the minor allele T of the rs2981578 SNP in the *FGFR2* gene impacts gene expression, reducing the potential growth of the mandible and, consequently, leading to MR.

The rs4752566, rs10736303, rs11200014, rs1078806, rs1219648 and rs2162540 SNPs were chosen for this study and showed an association with MR in the haplotype analysis. To the best of our knowledge, this is the first study that shows the impact of rs4752566 and rs1219648 SNPs on skeletal malocclusion. The rs4752566 SNP, located in intron 9 of the *FGFR2* gene, was previously linked with cleft lip [32], but its specific function remains unclear. The rs1219648 SNP is located in intron 2, as well as rs11200014, rs10736303, rs1078806, rs2162540 and rs2981578 and were previously associated with skeletal malocclusion [17]. There is speculation that genetic variations in intron 2 of the FGFR2 gene may be involved in enhancing histone acetylation and motif changing [33]. These molecular events corroborate the hypothesis that these SNPs modify the *FGFR2* expression and influence mandible development. Such regulatory effects at the intronic level also highlight the complexity of gene–phenotype interactions, suggesting that non‐coding polymorphisms may have significant biological influence in several signalling molecular networks.

One of the study's limitations is the use of a convenience sample due to the inclusion of only those patients who actively seek orthodontic treatment services. This limitation may introduce selection bias, leading to an unbalanced number of cases. In fact, the MR patients corresponded to 65% of the sample. Consequently, the results of this study may not be generalisable to the broader population. Future studies with population‐based sampling and multi‐centre recruitment are recommended to validate these associations and ensure representativeness across different ethnic and environmental backgrounds. Moreover, functional analyses to explore potential impacts of these variants on *FGFR2* mRNA structure or stability are encouraged for future investigations.

In conclusion, intronic SNPs in the *FGFR2* gene (rs4752566, rs10736303, rs11200014, rs1078806, rs1219648, rs2981578 and rs2162540) are associated with MR establishment and have the potential to serve as genetic biomarkers to early diagnosis and prediction of mandible growth.

## Author Contributions

Conceptualization: E.C.K. and C.K. Methodology: E.C.K. Software: C.L.B.R., and E.P.‐S. Formal analysis: C.L.B.R. Investigation: C.L.B.R., D.H., E.P.‐S., P.P., C.M.A. and S.B.‐M. Resources: E.C.K., C.K., P.P. and F.B.‐F. Data curation: C.L.B.R. and D.S.B.O. Writing – original draft preparation: C.L.B.R., C.M.A. and E.C.K. Writing – review and editing: all authors. Visualisation: C.L.B.R. Supervision: E.C.K., C.K. and S.B.‐M. Project administration: E.C.K. and C.K. Funding acquisition: E.C.K. and C.K.

## Funding

This study was supported by Alexander von Humboldt‐Stiftung (Küchler/Kirschneck accepted on 4 July 2019); Fundação de Amparo à Pesquisa do Estado de São Paulo (grant no. 2021/02704‐1); and Coordenação de Aperfeiçoamento de Pessoal de Nível Superior (Finance code 001).

## Ethics Statement

The sample analysed in this current cross‐sectional study was previously described by Kischneck et al. (2022) [7] as well as the sample size calculation. No additional patients were added to the sample after the initial study. The Human Ethics Committee at the University of Regensburg, Germany, granted approval for this study (reference number 19–1549‐101). All ethical guidelines outlined in the Helsinki Declaration were followed. Informed consent was obtained from all patients and their parents or legal guardians. Additionally, for patients under the age of 14, an age‐appropriate assent document was also utilised.

## Consent

The authors have nothing to report.

## Conflicts of Interest

The authors declare no conflicts of interest.

## Supporting information


**Table S1:** Raw data.
**Table S2:** Haplotype combinations.

## Data Availability

The data that supports the findings of this study are available in the Supporting Information of this article.
